# Parental Evaluation of a Responsive Parenting Program for Infants with Hearing Loss

**DOI:** 10.3390/children12010092

**Published:** 2025-01-15

**Authors:** Reinhild Glanemann, Karen Reichmuth, Stephanie Brinkheetker, Antoinette am Zehnhoff-Dinnesen, Katrin Neumann

**Affiliations:** Department of Phoniatrics and Pediatric Audiology, University Hospital Muenster, University of Muenster, 48149 Muenster, Germany; karen.reichmuth@ukmuenster.de (K.R.); stephanie.brinkheetker@ukmuenster.de (S.B.); am.zehnhoff@uni-muenster.de (A.a.Z.-D.); katrin.neumann@uni-muenster.de (K.N.)

**Keywords:** early intervention, family-centered early intervention, hearing loss, parental program, responsiveness

## Abstract

Background: Parental satisfaction is an important factor in the evaluation of early intervention programs but is rarely investigated. The Muenster Parental Program (MPP) is a short, evidence-based early intervention program that focuses on parental responsiveness. It is a family-centered intervention for parents of infants aged 3–18 months who have recently been diagnosed with hearing loss and fitted with hearing devices, including prior to or following cochlear implant surgery. Objective: We aim to receive feedback from parents regarding the process and outcomes of their participation in the MPP. Method: Following their participation, all participants of the MPP were asked to complete an evaluation questionnaire. This article reports feedback from the first 52 participants (44 mothers, 7 fathers, and 1 godmother). Their infants (*N* = 45) had moderate to complete hearing loss, they were aged 2–20 months, and 40% of them had an additional disease, disorder, and/or developmental delay. Results: Parents reported high levels of satisfaction with the content, didactics, setting, and individual benefits of the intervention, and a high recommendation rate (92%). The aspects most appreciated were meeting other affected parents and the concrete individual support of parent–child communication, including video feedback. Almost all parents (96%) reported a change in their communication style with their child. This confirms the results of a previous controlled intervention study on the enhancement of parental responsiveness via the MPP. Conclusions: This evaluation of the MPP from a parental point of view has revealed equally high satisfaction with the content, setting, and didactics amongst all parents regardless of any potentially influential parent or child variables. The MPP is well suited to a wide range of close caregivers’ needs despite the known diversity of children with hearing loss and their parents or families.

## 1. Introduction

### 1.1. Effective Early Intervention Following Newborn Hearing Screening

The implementation of Newborn Hearing Screening (NHS) programs has resulted in the identification of childhood hearing loss (HL) (the term “hearing loss” (HL) is used in this paper to denote all grades of hearing impairment as recommended in the *World Report on Hearing* of the WHO [1]) and treatment with amplification and enrolment in early intervention services at a younger age in many countries [2]. Following the recommendation of the Joint Committee on Infant Hearing [3,4], screening of HL should be completed by at least the age of one month, audiological diagnosis by the age of three months, and enrolment in early intervention services by the age of six months (Early Hearing Detection and Intervention Guidelines—EHDI 1-3-6). A systematic review [5] of the outcomes of NHS programs revealed better performance in speech, language, and literacy. The chances of children even attaining age-appropriate language performance, regardless of their grade of HL, has increased [5,6,7,8,9].

The implementation of early intervention services is still too often conceptualized without any professional therapeutic parental support for the development of spoken language [10]. The provision of technical hearing devices alone as a concept of early intervention is not sufficient. Despite the positive trend of earlier detection, the variability of results pertaining to language performance remains large [6,7,8,11]. Current expert opinion and guidelines consider the additional initiation of family-centered early intervention (FCEI) a crucial factor in optimizing child development [1,10,12,13,14,15].

The core idea of family-centered approaches is to support parents in taking a central role in promoting their child’s development by reinforcing their intuitive parental abilities and resources [13,14,16]. The younger the age of the child at diagnosis, the more parents believe in their own ability to influence their child’s development [17]. This fact positively goes hand in hand with findings underscoring the great impact that the quality of parent–child interaction and the quality of parental language input have on language and social–emotional development [18,19,20,21,22]. Supporting caregivers in developing a responsive and dialogic parenting style should therefore be an inevitable component of FCEI for infants with HL; it promotes the parents’ communication with their child in every life experience [16,20,23,24]. Consequently, concepts of FCEI programs must be tailored to the special needs of parents of infants with all grades of HL. The means of intervention used should be evidence-based [13,14].

A systematic review by [25] revealed that it is only within recent years that parent training programs that have undergone controlled and prospective research to investigate their effectiveness for children with HL have been published. Their review identified five evidence-based parent training programs that aim to empower parent–child communication in this target group. Two of these programs already work with parents of infants in their first months of life, namely the Muenster Parental Program (MPP) [26,27] and Parent-Implemented Communication Treatment [28].

Although the effectiveness of interventions is increasingly the focus of research, parental satisfaction with an intervention is still rarely considered. This is despite the fact that parental satisfaction has been shown to be yet another meaningful factor in the evaluation process of FCEI [17,29,30,31]. Gascon et al. [29] argued that, in addition to the use of objective measures of behavioral change in parents and in children’s outcomes, the effectiveness of an early intervention program should also be assessed by the parents themselves, who naturally differ from each other in their individual beliefs and preferences.

### 1.2. Situations, Needs, and Perspectives of Parents

Approximately 95% of children with HL have typically hearing parents [31]. These parents are hit by the unexpected news of their child’s diagnosis relatively soon after the child’s birth. What are the needs of such parents in this life situation? Several studies have examined the parental perception of the process of diagnosing HL after NHS, including all information and support they received [29,32,33,34]. These studies revealed that parents’ emotional and perceptual experiences in this unique situation are diverse and complex. Many parents are overwhelmed by worry and grief, are often in a state of shock, and may be in denial about the diagnosis. Unsurprisingly, parents describe this early stage around and after diagnosis as difficult and emotionally stressful. But parents quite often also feel a strong desire for action.

The research suggests that parents wish to have prompt pediatric audiological support, including relevant information about HL and hearing devices. They also express a great need for early FCEI in order to cope with the new situation and to help them to support their child. If access to such services is delayed or not provided at all, parents perceive this as a loss of the advantage offered by early detection of HL [32]. Parents judge an early intervention as particularly helpful if it focuses on everyday parent–child interaction within the family [29,30,34]. They desire concrete guidance on how to implement the advice they receive regarding communication with their child within their daily lives [30].

In cases of profound or total HL, parents carry the additional burden of making a decision concerning communication mode and/or cochlear implant (CI) surgery. On the one hand, provision with CI(s) is perceived as a benefit for their child’s hearing and spoken language development; on the other hand, the idea of their child undergoing surgery elicits fear in nearly all parents [35]. About 40% of children with permanent HL present with additional impairments [36]. These can be diseases, syndromes, or other developmental risk factors. This is unquestionably a further strain on the child and can make parents even more insecure about the child’s developmental chances in life. They often feel helpless and worry about how to handle their child and communicate with them [37].

Besides the specific and individualized help provided by professionals, parents appreciate the social and emotional support given by their spouse, other family members, and friends [33,38,39,40]. Parents also wish to connect and exchange with other parents who are in the same situation [13,14,33,41].

FCEI concepts must carefully consider all of these aspects and parental needs. Only then is such intervention able to empower parents to regain confidence in their parenting skills. Parents play a crucial supporting role in the learning processes of their child, which begin immediately after birth and of which early preverbal dialogs are an important part [42,43].

Parental needs were taken into account in the development of the MPP. The following section presents an overview of the aims, concept, setting, methodology, and content of this evidence-based parent training program.

### 1.3. Fulfilling Parental Needs: Muenster Parental Program (MPP)

The Muenster Parental Program (MPP) for parents of infants with HL is a short FCEI, designed to be offered to parents early after the diagnosis of HL following NHS. It addresses the parents of infants with all grades of HL, including those who also have additional developmental disorders or delays. The concept [27] is specifically tailored to the needs of parents of infants with HL within the first 18 months of life. A characteristic of the MPP is that it covers a wide range of children’s preverbal development, even in cases of delayed diagnosis of HL. It is conceptualized as one module of a comprehensive and interdisciplinary early intervention service. The MPP is carried out by early interventionists, who are specifically certified for this program. It is also suitable for parents in the period prior to and following cochlear implant surgery as a part of the habilitation process.

The MPP aims to empower parents in their use of conducive, responsive communication behavior in order to support their child’s speech, language, and hearing development in everyday situations from early life onwards. Mothers and fathers (or other close caregivers) are invited to participate as a pair. The program also offers the possibility to connect and exchange information with other affected parents early after diagnosis with the aim of reducing parental stress and the emotional burden carried at that time. The MPP is designed to prevent or reduce an overly directive parenting style by strengthening parents’ intuitive responsiveness as soon as possible after the diagnosis of HL.

The MPP combines child-free parental group sessions with single sessions for each participating parent and their child. It thereby provides space for exchange between parents as well as for the individual needs of each parent–child dyad. The program was developed for small groups of four to six families and comprises eight sessions, usually running on a weekly basis, including six group and two single sessions. It is flanked by two individual counseling sessions for each family. When the child reaches 24–30 months of age, a single refresher session is also offered to parents. In this refresher, parents are supported in being responsive to the (in most cases) now verbal child via dialogic picture book reading, as recommended, for example, by DesJardin et al. [24].

The curriculum of the MPP (see [27] for details) follows the same basic principles as responsive parenting programs for hearing children with other developmental risk factors (for an overview, see [44]). These are often based on the methodology of the well-known Canadian Hanen Early Language Program It Takes Two to Talk for late talkers [45]. Their principles were adapted and expanded for the MPP in order to cater to the needs of parents of infants with HL, largely on the basis of the Natural Auditory Oral Approach for children of this target group (e.g., [46]).

In preverbal dialogs, parents normally adjust their interactive behavior intuitively to the infant’s limited repertoire by parsing, exaggerating, and repeating while concentrating on the infant’s focus of attention [42]. In the group sessions, the MPP makes parents aware of these aspects of responsiveness and links them to guiding principles and images that are easy to grasp (e.g., “Take turns with your child”—with a seesaw as a pictorial metaphor). Parents learn four key aspects of responsiveness and their application in daily interaction, such as playing and communicating with their child. Video-recorded examples of other parents using responsive parenting serve as models to enhance the theoretical content and bring the theory to life. In the single sessions, parents are then supported in transferring and individualizing the learned hallmarks of a successful parent–child interaction to their own lives. Here, video feedback is the core didactic component used to promptly confirm the parent in their use of responsiveness.

Two approaches have been taken in evaluating the MPP. One is the use of prospective and controlled intervention studies on outcomes. One example of this was a video-based interaction analysis performed before and after participation, which revealed more communication-enhancing strategies (responsiveness) and fewer communication-inhibiting behaviors in the intervention group than in the control group [26]. In this intervention study, the infants who all communicated on a preverbal level (mean age 6.8 months) showed a greater increase in vocalizing behavior after participation in the MPP than infants of parents in the control group.

The second line of evaluation, which is reported in this paper, consists of obtaining subjective feedback from parents who had just completed the program. This assessment of their satisfaction with the MPP and perceived individual benefit from their participation is an integral part of the program. The questionnaire thereby serves to provide quality assurance regarding the MPP, both for research contexts and for practice.

The present study aims to answer the following research questions:

- How do parents assess the MPP with respect to content, didactics, and setting?
- How do parents assess the quantitative and qualitative impact of the MPP on their own communication behavior and that of their child?
- Does the MPP satisfy the needs of all participating parents equally even though they and their children differ in the following variables?
  − Parent-related variables: sex, academic status, HL in a close family, attendance as a couple, and dealing with the option of cochlear implant surgery;
  − Child-related variables: type (unilateral or bilateral) and grade of HL, presence of an additional condition, and birth-order position.

We hypothesized that parents would view the program positively regarding both its objectives and actual outcomes, that they would perceive improvement and greater confidence in communicating with their child, and that they would report that the benefits outweighed the cost and time invested. We expected parent and child variables to have only a minor impact on these outcomes, a hopeful consequence of the fact that one important aim during the development of the MPP was to serve the large heterogeneity of these variables within the target group. There was, however, some uncertainty about whether parents of children with severe developmental delays or disorders would be as satisfied as other parents.

## 2. Materials and Methods

### 2.1. Participants

The socio-demographic data of the participants and their infants are shown in Table 1. All had participated in MPP groups conducted in the authors’ clinic, a tertiary center for phoniatrics and pediatric audiology. The data of two parents had to be excluded from the analysis due to insufficient proficiency in written German. This resulted in a total of 52 participants, comprising 44 mothers, 7 fathers, and 1 godmother. The average number of participants per group was four. Eight families within the sample (thereof 11 participants) were bilingual or trilingual. They spoke one or two languages in addition to German, namely Russian, Polish, Arabic, and/or Turkish, and German Sign Language in one case. Approximately one-third of parents had a university degree.

All but one of the participants’ children were identified by NHS within their first month of life; the remaining child was not identified until his 11th month because he had suffered from a life-threatening condition requiring intensive care before his hearing screening could take place. Sixty percent of the children (27 children) had no additional diseases that might affect child development. The remaining 18 children had an additional disease or developmental delay or both (four cases of cytomegalovirus infection, three cases of Down syndrome, three cases of developmental delay of unknown cause, and one case each of prematurity, hypophyseal dysfunction, peripartum asphyxia, prenatal stroke, CHARGE syndrome, Distal 18q- (De Grouchy syndrome 2), Pallister–Killian syndrome, and Smith–Magenis syndrome).

### 2.2. Parental Questionnaire for Assessing the Muenster Parental Program (Q-MPP)

Parental feedback on the MPP was obtained using the self-developed Q-MPP (Parental Questionnaire for assessing the Muenster Parental Program). This instrument evaluates the content, didactics, and setting of the MPP. It also asks parents whether their own and/or their child’s communication behavior has changed after having participated in the MPP. The Q-MPP comprises 31 questions in total, of which 21 are process-related questions (items 1–5 d and 7–18; see Table 2), 6 are outcome-related questions (items 6a-b and 19a-20b), and 4 are questions related to general aspects of the MPP (items 21–24). Of all questions, 23 use a Likert item response scale (0 = strongly disagree, 1 = disagree, 2 = neither agree nor disagree, 3 = agree, 4 = strongly agree), 3 are yes/no questions, and 5 are open-ended questions. In the introductory text of the Q-MPP, parents are explicitly encouraged to bring up positive as well as negative aspects in their feedback.

The Q-MPP was validated with reference to the parent version of the standardized FBB-E (Fragebogen zur Zufriedenheit mit der Behandlung-Eltern) [47], which is a questionnaire on parental satisfaction with family-centered child treatment in psychiatric and psychotherapeutic interventions. The first 29 participants of the MPP completed both the Q-MPP and the FBB-E. Comparing the mean total score of the FBB with the mean total score of the scaled questions of the Q-MPP yielded a strong correlation of r = 0.64 and *p* = 0.01 (Spearman’s rho) (see [48] for details).

### 2.3. Data Collection

At the end of the final group session, a colleague who was not involved in the MPP handed out the Q-MPP to those parents who attended in full and collected it after completion. Full attendance was defined as having attended at least four of the six group sessions and both individual sessions. On average, each participant reported here missed one group session. In order to obtain unbiased responses, the professional leading the group was not present while parents completed the questionnaire, and parents were informed that the MPP-group leader would not see the completed questionnaires. After the questionnaires were returned, parents had the opportunity to provide oral feedback within the group with the MPP-group leader present.

### 2.4. Data Analysis

The “overall satisfaction” variable was calculated as the mean of the total scores of all Likert-scaled questions. Normal distribution of these mean values was verified by the Kolmogorov–Smirnov test. The effect of parent and child characteristics on overall satisfaction was analyzed using independent *t*-tests and Pearson correlation for grouped variables and metric variables, respectively.

All answers to open-ended questions were assigned codes by a rater who did not participate in the MPP groups and who was blinded to the participants. These codes were labels or descriptions of the content of the participants’ answers to a specific question. The codes were then sorted into categories (groups of similar codes). The reliability analysis was performed by randomly selecting 20% of all responses to the open-ended questions, the codes and code descriptions of which were then given to a second rater who was blinded to participants. The reliability analysis returned a Cohen’s kappa of 0.84, *p* < 0.001. Differences in coding were discussed by the two raters and adjusted where applicable. MAXQDA 2022 (VERBI—Software 2021, Berlin, Germany) and SPSS software (SPSS Statistics for Windows, Version 25.0, Fa. IBM Corp., Armonk, NY, USA) were used for these analyses.

## 3. Results

The response rate for the Q-MPP was 100%.

### 3.1. Overall Satisfaction with Content, Didactics, and Setting

Each of the 23 Likert items was answered by at least 43 parents (median = 51, Table 2). A mean score of 3.2 (SD 0.5) (maximum possible score = 4) was obtained, which indicated high levels of overall satisfaction with the program.

**Table 2:** Results of the Likert-scaled items of the Q-MPP (0 = strongly disagree, 4 = strongly agree).

Item	*N*	*M*	*SD*	*Min*	*Max*
I found it helpful to meet parents of other children with hearing loss.	50	3.8	0.5	2	4
I found it helpful to watch videos of other children and their parents.	52	3.4	0.7	2	4
I found the information given in the short presentations on child development helpful.	52	3.5	0.7	1	4
I find the clock, mirror, seesaw and watering can figures helpful to remember the four principles of promoting communication.	52	2.9	1.1	1	4
I find the four principles of promoting communication helpful in communicating with my child. Take your time to observe! (clock) Imitate and follow your child! (mirror) Take turns with your child! (seesaw) Let your own experience flow in! (watering can)	48 48 48 49	3.5 3.5 3.4 3.3	0.7 0.6 0.7 0.8	2 2 2 1	4 4 4 4
Regarding the four principles of promoting communication: I put them into practice with my child every day. I find them difficult to apply.	51 44	3.3 1.3	0.6 1.1	2 0	4 4
I found the games that were played to introduce the different topics helpful (e.g., the torch game, shopping without understanding the local language, the climbing caterpillar, strange sounds in the jungle)	52	3.0	0.9	0	4
I found the conversations within the single sessions helpful.	52	3.4	0.6	2	4
I found being filmed within the single sessions helpful (video feedback).	52	3.3	0.9	0	4
I found the single sessions helpful in applying the contents of the group sessions to communication with my child.	52	3.4	0.8	1	4
I find the suggestions for ‘Accompanying my child in learning to listen within everyday life’ helpful.	49	3.2	0.7	2	4
I find the toy materials that were presented helpful in my daily routines with my child.	52	3.2	0.8	1	4
I found the topic ‘Communication when looking at picture books’ helpful.	52	3.4	0.7	2	4
I find the written descriptions in my parents’ manual helpful.	52	3.2	0.9	1	4
I found the homework tasks helpful.	51	2.5	1.0	0	4
Regarding the topic ‘accepting my child’s disability’: I found the content helpful. It was given sufficient time.	49 49	2.9 3.1	1.0 0.9	0 1	4 4
I found the language (terms, choice of words, foreign words) used by the group leader easy to understand.	51	3.7	0.6	2	4
I felt comfortable in the facilities.	52	3.5	0.6	2	4

Note: the values for Item 6b were inverted in order to calculate the mean score.

**Figure 1:**
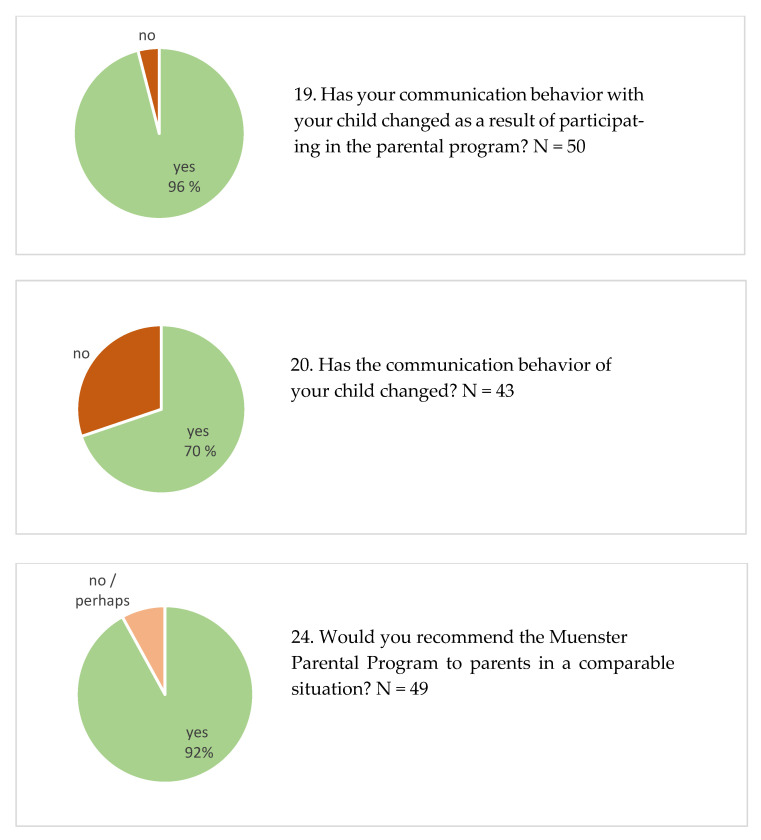
Percentage scores given by valid answers to the yes/no questions in the Q-MPP.

Ninety-two percent of participants reported that they would recommend the MPP to parents in a comparable situation (Figure 1, Item 24). Table 3 illustrates the aspects that participants most liked, considered redundant, or felt were lacking in the MPP. All response categories that received at least three votes are reported in Table 3.

No significant effect was found for participant variables (gender, academic status, attendance as a couple, and HL within the close family) or child variables (type and grade of HL, presence of an additional condition, and birth-order position) on the overall satisfaction measure. Group means of this measure differed only marginally by a (non-significant) maximum of 0.14.

### 3.2. Impact on Parent–Child Communication

Ninety-six percent of participants reported behavioral changes in their communication with their child as a result of participating in the MPP (Figure 1, Item 19). The categories used for parents’ descriptions of these changes are listed in Table 3, Item 19a. Seventy percent of participants also reported differences in the communication behavior of their child during the course of the MPP (Figure 1, Item 20). The changes they perceived are shown in Table 3, Item 20a.

## 4. Discussion

The MPP is a responsive parenting program designed for parents with infants aged 3–18 months who have recently been diagnosed with HL. This questionnaire-based study examined parental satisfaction with the MPP and the perception of individual benefits regarding parent–child communication after participating in it.

Parents were asked to give their feedback by means of the Q-MPP questionnaire. This questionnaire has already proven to be an adequate feedback instrument for the quality management of the MPP [48] and is an integral part of it. The Q-MPP enables ongoing evaluation of the intervention from a parental point of view, a practice recommended by, for example, the international consensus statement for best practice in FCEI [13].

The present study complements previous prospective and controlled research into the effectiveness of the MPP [26]. The MPP is, to the best of the authors’ knowledge, the only responsive parenting program for parents of a child with HL that has reported systematically compiled parental feedback so far. In this respect, this study enhances the small volume of data on parental satisfaction within this field [31].

### 4.1. Parental Satisfaction with Content, Didactics, and Setting

Overall, the parental evaluation described here has revealed a high level of satisfaction with the content, didactics, setting, and individual benefit obtained following the intervention. Despite the high level of organizational effort some families required in order to participate in the weekly MPP appointments, nearly all parents (92%) would recommend the MPP to other parents in a comparable life situation. Due to the large catchment area of the clinic, more than half of the parents traveled further than 50 km each way (and some even more than 100 km) to attend each of the weekly sessions of the MPP over two months. This commitment reflects parents’ real desire for support in the new and challenging life situation of bringing up an infant with HL.

When parents were asked about the most valuable content within the MPP, the two most common answers given were meeting other affected parents in the group, and the single sessions with individual video feedback. These were closely followed by concrete individual support in communicating with their child and the use of video demonstrations in the group sessions. The latter shows other parents interacting responsively with their child with HL as a method of video-supported imparting of theoretical content. Taken together, these specific answers emphasize the value of having both single and group sessions within the MPP.

The above aspects of participants’ feedback reflect Jackson [33] and Moeller et al. [13,14] in demonstrating that exchange with other affected parents should already be a fundamental part of FCEI following NHS. The MPP offers parent-to-parent support in the sense of a support group, as described and strongly recommended by Luterman [49]. When parents start the MPP, they are all “newcomers” in a comparable, challenging life situation, and they are able to support each other.

The positive assessment of the concrete support provided for parents’ individual communication with their child corresponds closely to the findings of Decker and Valloton [30] in their interview study. They reported that parents want to receive concrete advice on how to interact with their child in daily communication, instead of just being told to talk to their child as often as possible. Parents receive the latter advice very often from professionals [30], but it has been found to be an insufficient technique for supporting language acquisition in children with HL [18]. In contrast, video feedback is known to be a very effective means of FCEI in enhancing parental responsiveness [50]. Providing mothers with prompt feedback on their responsive actions is recommended in order to reduce feelings of insecurity about responding adequately to the expressions and emotional needs of their child with HL [20]. It can therefore be assumed that, although video feedback usually provokes skepticism in parents at first, they particularly appreciate this method as a concrete help in the MPP.

The parents of children with additional conditions judged the video feedback just as positively as all other parents. This finding supports the strong recommendation made by Provenzi et al. [37] to use video feedback in responsive parenting programs for families of children with (neuro)developmental disabilities.

Parents also appreciated the use of video demonstrations of other parents’ parent–child communication in the group sessions. This type of indirect parent-to-parent support is known to provide relief to parents because of the positive effects of experiencing successes in responsive parenting and the lived reality of other affected parents in a similar life situation [50]. In such cases, parents appreciate that they learn from other parents, rather than professionals, about how to succeed in a responsive parenting style.

Two-thirds of parents stated that they did not feel there was any content missing from the MPP. This further confirms the high level of parental satisfaction with the course. When asked about redundant aspects of the MPP, 23% of parents responded that some aspects of the content could have been imparted in a more straightforward manner. This notion was mainly reported by parents with academic levels of education. The concept of the MPP, especially in the group sessions, however, intentionally encompasses a wide variety of didactic techniques from adult education in order to engage with parents with different preferred learning styles and also various levels of language comprehension. The MPP-group leader tailors the intervention more to the individual needs of the parent within the two single sessions.

### 4.2. Altered Parental Communication Behavior

The MPP effectiveness study by Glanemann et al. [26] demonstrated that the combination of video-supported theoretical content with practical application supported by video feedback enhanced parents’ responsiveness significantly. In line with this, nearly all parents within the present study state that their communication behavior towards their child had changed. When asked to describe this change, parents almost exclusively reported key features of responsive behavior. Taking the findings of these two studies together, parents are not only more responsive following the MPP but also more aware of their increased responsiveness. Knowing about strategies to promote communication, together with awareness of actually using these strategies, enables parents to experience self-efficacy. This again is an important personal resource in coping with the situation of having a child with a disability [17,51]. Increased self-efficacy is associated with higher parental well-being and mental health and reduces the risk of depression [51]. The strengthening of self-efficacy is therefore considered a crucial component of resource-oriented working with parents [16,17,51]. We did not assess self-efficacy and well-being explicitly in the Q-MPP, but it may be assumed that the change parents experienced in their own communication behavior has a positive impact on their perceived self-efficacy.

Some parents also were explicit about the related effect of feeling empowered in their parenting role. This is a key aim of the MPP. The following answers to open-ended questions aptly express the security that parents felt they had gained and their increased self-trust in being with their child, such as, “I now feel more confident in everyday interaction and play with my child“, and, “we no longer feel alone in coping and have regained control”. Some parents also reported that they experienced more joy in interacting, which may be an additional sign of more parental well-being.

A recent meta-analysis has shown that responsive parenting combined with highly effective facilitative language strategies has a large impact on language acquisition in children with HL and has called for more early parental support on this aspect [21]. Although the MPP is primarily for families who have a preverbal infant, it already prepares parents for the task of promoting their child’s later speech–language and listening skills, at the transition to the verbal stage. In the final MPP group session, parents learn to transfer responsiveness to dialogic picture book reading, combined with highly facilitative language strategies. Parents appreciated this topic, even if some children were still too young to look at picture books. In this way, parents are already prepared at this early stage for the importance of dialogical picture book reading, which is deepened in the refresher session after the child’s second birthday (see Section 1.3).

### 4.3. Improved Child Communication Behavior

Seventy percent of the parents describe that, following the MPP, their child is more attentive, vocalizing and imitating more than before. This change in communication behavior reflects an important step in their speech–language development. Although such progress is to be expected at this age, an earlier study found more strongly increased levels of child vocalization in MPP participants than in a control group [26].

### 4.4. Influence of Parent- and Child-Related Variables on Parental Satisfaction with the MPP

There was considerable heterogeneity with respect to the many parent- and child-related variables in the study group that could impact upon the present evaluation of the MPP. One important result is that the intervention was effective in reaching parents from across the spectrum of school and academic degrees. Whilst the results show that the concept of the MPP can cope with great heterogeneity, the subgroups of fathers, couples, parents of children with profound or total HL, and parents of children with additional diseases and conditions deserve more detailed examination.

#### 4.4.1. Participation of Fathers and Couples

Most participants within our study group were mothers. This is typical of most early intervention settings [52]. Fourteen percent of our subgroup were fathers. This is in line with Hintermair and Sarimski’s [40] finding that 14% of fathers in Germany frequently attend one appointment for early intervention and a subgroup of 11% attends all such appointments. We presume that more fathers would wish to participate in the program but are faced with logistic problems.

Paternal needs are increasingly given attention in current research [38,39,40,52]. In our study, nearly all fathers who participated did so together with their partners. This corresponds to the finding that fathers generally prefer joint participation over individual participation [53]. There is a need for more FCEI to address couples, in order to empower not only their individual skills but also their complementary resources [38]. The MPP meets this need. By involving couples, it also enables fathers and mothers to strengthen their naturally different parental behaviors with their child [54].

The consistently high levels of overall satisfaction, regardless of gender and participation status (single or pair), demonstrate that the concept of the MPP reaches mothers, fathers, and couples equally. In one case, the couple consisted of a mother and godmother, a constellation whose participation is seen as beneficial by, for example, Jackson [33], who emphasizes that, in addition to parents, other close reference persons, such as godmothers, may be included in FCEI.

Participating as a couple or pair, however, often poses an organizational challenge for a family. It generally helps families if an intervention is adapted to time periods outside the working hours of the fathers [38,39,40]. In addition to the conflict between work and early intervention, families must organize someone to look after the child(ren) of the family. Two participants withdrew from the MPP during the initial phases, and some further cancelations of single appointments occurred, all due to organizational difficulties. This reflects the fact that needs and feasibility are not the same thing.

#### 4.4.2. Parents of Children with Profound or Total Hearing Loss

In the present study, more than a third of children had profound-to-total HL at the beginning of the MPP, a diagnosis that poses a considerable risk to their language development and therefore often heavily burdens parents, as described in detail in Section 1.2. This could be responsible for the high proportion of parents of this subgroup participating in the MPP.

In cases where cochlear implantation is planned, Bruin [23] and Yoshinaga-Itano et al. [22] urgently recommend family support prior to the surgery in order to bridge the time until the operation in a meaningful way. This time is stressful for the parents, and family support is precious because an empowered family already serves as a solid basis for child learning even prior to this specific audiological intervention [23]. Bruin [23] has described the MPP as a good example of such family support.

#### 4.4.3. Parents of Children with Additional Diseases and Conditions

Forty percent of the children in the present sample had an additional disease or developmental delay in addition to their HL. Parental sorrow and grief may be assumed to be larger in this subgroup than in parents who are “only” confronted with the diagnosis of HL. However, the overall parental satisfaction level was as high as for parents of infants without additional conditions. This may be attributed to the fact that the MPP’s concept is particularly geared toward fostering parental responsiveness in the pre-intentional and pre-symbolic developmental stage of the child.

### 4.5. Coping with the Disability

Hintermair [51] emphasized that coping with the HL of their child is a substantial burden for parents at the early stage after diagnosis. The MPP concept provides time, parent-to-parent support in the group, and a theoretical model as a framework for coping with grief [55]. However, the parents’ feedback leaves the question open of whether some would have liked to explore the topic of grief in more depth, in another way, or maybe not at all. The MPP-group leaders noticed that parents delved into this topic to different depths within the group sessions. These different levels of engagement and handling of the situation reflect the diverse ways in which people are known to respond to a serious medical diagnosis [34]. The group leaders also reported varying feedback regarding the use of the theoretical grief model. Some participants reported that they were better able to understand their partner’s or family member’s feelings afterwards, as they were probably at a different stage of grief than themselves. Other participants did not talk about their thoughts on the model in the group at all.

The grief associated with the diagnosis certainly cannot be conclusively overcome within a short intervention. It will accompany the parents throughout the following months and years. One positive effect of the MPP is that almost one-third of parents remain in regular contact with each other of their own accord after completion and, in this way, a form of private self-help group develops. If, however, parents present strong symptoms of stress during the MPP period, they are informed about the possibility of receiving professional psychotherapeutic support.

### 4.6. Parental Wish for Immediate Support

Parents desire immediate support following the diagnosis of an HL via UNHS, as reported in Section 1.2. Experts underline the urgency of this parental desire [13,14], which can be met through the consistent implementation of the EHDI 1-3-6 criteria [3,4] (see Section 1.1). Encouragingly, counting the supply of hearing devices as a specific initial early intervention suggested that these criteria were fulfilled for nearly 70% of the infants in the present study. Unfortunately, the additional benchmark of enrolment in family-centered intervention within the first six months of life (see Section 1.1) was only met in approx. 30% of children in the present study. The latter, more extended understanding of early intervention should be the rule according to Smith et al. [10] and the international consensus statements on FCEI [13,14].

The present data, as well as data from other German regions [20], underline the urgent need for the implementation of earlier enrolment in FCEI following the diagnosis of an HL in Germany. In other highly developed countries, the provision of hearing devices at an early stage, combined with enrolment in FCEI (which includes parental support), has been found to lead to better outcomes regarding child development [2,5,8,22]. A recent German guideline on evidence-based practice also strongly supports this recommendation [15].

### 4.7. Measuring Parental Satisfaction Serves Quality Assurance of Early Intervention

In addition to proving the effectiveness of the MPP [26], the present survey of parental satisfaction fulfills the further FCEI quality assurance requirement of international consent, which has been provided by the international consensus statements of FCEI [13,56].

A third FCEI requirement is to ensure the qualification of early interventionists [57]. The MPP professional training, provided by the authors since 2012, meets this requirement. So far, it has enabled parents in nine of sixteen German federal states to join an MPP group relatively close to their place of residence. There, too, the Q-MPP serves quality assurance as an integral part of the program.

### 4.8. Limitations

One limitation of this study is the small sample size of the study group. Accordingly, the analysis of the effects of parental subgroups, such as gender, participation as a couple, degree of the child’s HL, and parental education could only be exploratory. It would also be interesting to receive feedback regarding long-term effects from parents not only directly after participation but also at a later time point.

A future version of the Q-MPP should include the assessment of self-efficacy more explicitly as well as the parental perception of their involvement in the program. These are essential parameters for the evaluation of FCEI programs [17].

Even though parents are very satisfied with the MPP, further adaptations for families with a child who has an additional severe developmental disorder or delay are necessary from a therapeutic perspective. These parents may, for example, benefit from watching video clips not yet included in the training course which show interaction with severely affected children. It would also be beneficial to adapt the MPP concept for families of these children by incorporating methods of augmentative and alternative communication.

## 5. Conclusions

Parental satisfaction is an important component of the evaluation and quality assurance process of early intervention programs. A program’s effectiveness should be evaluated not only by the providers themselves but also by participating parents who naturally differ in their individual beliefs and preferences. The questionnaire-based feedback study reported here meets this objective: the results reveal a high level of overall parental satisfaction with the content, didactics, setting, and individual benefits following the intervention. This means that the MPP is well suited to a wide range of close caregivers’ needs despite the known diversity of children with HL and their parents or families.

In summary, the MPP meets mothers’ and fathers’ needs for immediate and individualized support not only in coping with their new life situation shortly after the diagnosis of HL but also with their desire to share their experience with other affected parents. Parental assessments confirm that the MPP is beneficial for infants with all types of hearing devices, including those prior to or following cochlear implant surgery.

## Figures and Tables

**Table 1:** Characteristics of participants (parents or carers) and their children.

**A. Parents or carers (*N* = 52)**	***N***	**%**	***M***	***SD***
Relationship with the child Mothers Fathers Godmother	44 7 1	85 13 2		
Participating in pairs (*N* = 6 pairs) Mother & father Mother & godmother	10 2	19 4		
Age in years			34.3	5.2
First language German Other	46 6	88 12		
Family language German only German + 1 or 2 spoken languages German + sign language	41 10 1	79 19 2		
Education Academic Non-academic	16 36	31 69		
Hearing status (grade of HL ^1^) Mild-to-severe HL (wearing HA) thereof unilateral HL Profound HL (wearing CI and/or HA)	3 1 1	6 2 2		
Close family member with HL	10	22		
Distance from home to MPP center <50 km 50–100 km >100 km	24 24 4	46 46 8		
**B. Children (*N* = 45)**	***N***	**%**	***M***	***SD***
Sex Girls Boys	25 20	56 44		
Age in months at start of MPP (range: 2–20, median: 8)			9.4	4.9
Birth-order position Firstborn Subsequent sibling	25 20	56 44		
Identification of HL by NHS Yes, within 1st month No	44 1	98 2		
Age at diagnosis (months, median: 3)			3.4	3.1
Age at HA fitting (months, median: 3)			4.0	3.1
EHDI 1-3-6 guidelines fulfilled	31	69		
Age at beginning of therapeutic or pedagogic early intervention (MPP or other) (months) ≤6 months 7–9 months 10–12 months >12 months	23 9 7 6	51 20 16 13		
Grade of HL ^1^, bilateral (better ear) Moderate (35–49 dB) Moderately-severe (50–64 dB) Severe (65–79 dB) Profound (80–94 dB) total (>95 dB) Unilateral	8 7 8 2 14 6	18 16 18 4 31 13		
Grade of HL ^1^, unilateral Moderate (35–49 dB) Moderately-severe (50–64 dB) Severe (65–79 dB)	2 1 3	4 2 7		
Type of amplification prescribed Acoustic hearing aid (HA) Bone-conduction HA Cochlear implant (CI) CI & HA	34 5 4 2	76 11 9 4		
HL threshold fulfilling the criteria for CI at beginning of MPP	14	31		
Cause of HL Genetic ^2^ Acquired ^3^ Unknown	18 7 20	40 16 44		
Children with additional disease, disorder and/or developmental delay Mild to moderate Severe Profound	8 6 4	18 13 9		

^1^ Grades of HL as recommended by WHO [1], based on an average hearing threshold (dB HL) at 500, 1000, 2000, and 4000 Hz for the better-hearing ear. ^2^ familial HL, non-syndromic anomalies of the external ear, and syndromes such as Down, Pallister–Killian, Distal 18q- (De Grouchy syndrome 2), and CHARGE. ^3^ cytomegalovirus, postpartum hyperbilirubinemia, peripartum asphyxia, prenatal stroke. *Abbreviations*: CI = cochlear implant; HA = hearing aid; HL = hearing loss; EHDI = Early Hearing Detection and Intervention Guidelines.

**Table 3:** Categories applied and total numbers and percentages of answers to open-ended questions in the Q-MPP.

	*N*	% of 52
19a. How has YOUR communication behavior with your child changed?		
I communicate more consciously/intensely/effectively.	30	58
I let my child take the lead/I imitate my child.	14	27
I observe more, wait longer, am more attentive.	13	25
I take more time in general.	4	8
I am more joyful/happier within the communication.	3	6
I apply the four principles.	3	6
20a. How has the communication behavior of YOUR CHILD changed?		
She/he shows more interest/attentiveness/eye contact.	10	19
She/he vocalizes/babbles (more).	12	23
She/he imitates more often, is successful in turn-taking.	9	17
She/he is (more) joyful.	9	17
21. What did you like most about the MPP? Please name 1–2 issues		
Meeting other affected parents	32	62
Single sessions with video feedback	15	29
Guidance on communicating with my child	11	21
Video demonstrations in group sessions	10	19
Expertise/information on my child’s hearing and/or speech-language problem	7	13
Presentation of games/books	7	13
Didactics/introductory games	6	12
Everything	5	10
Empowerment	3	6
22. What did you consider redundant in the MPP?		
“Nothing“ or no entry	22	42
Suggestions to reduce time spent on various content	12	23
23. What did you feel was lacking from the MPP?		
“Nothing“ or no entry	35	67
Time for more exchange	6	12
More single sessions	3	6

## Data Availability

The dataset is available upon request from the authors.

## Abbreviations

CI = cochlear implant; EHDI = Early Hearing Detection and Intervention Guidelines; FBB-E = Fragebogen zur Zufriedenheit mit der Behandlung-Eltern; FCEI = family-centered early intervention; HA = hearing aid; HL = hearing loss; JCIH = Joint Committee on Infant Hearing; MPP = Muenster Parental Program; NHS = Newborn Hearing Screening; Q-MPP = Parental Questionnaire for assessing the Muenster Parental Program; UNHS = Universal Newborn Hearing Screening; WHO = World Health Organization.
